# Expedited re-design of multi-band passive microwave circuits using orthogonal scaling directions and gradient-based tuning

**DOI:** 10.1038/s41598-024-59512-7

**Published:** 2024-04-23

**Authors:** Slawomir Koziel, Anna Pietrenko-Dabrowska, Ubaid Ullah

**Affiliations:** 1 Engineering Optimization & Modeling Center, Reykjavik University, 102 Reykjavik, Iceland; 2 Faculty of Electronics, Telecommunications and Informatics, Gdansk University of Technology, 80-233, Gdansk, Poland; 3 Networks and Communication Engineering Department, Al Ain University, P.O. Box 112612, Abu Dhabi, United Arab Emirates

**Keywords:** Microwave circuits, Passive devices, Circuit re-design, Geometry scaling, EM-based optimization, Electrical and electronic engineering, Computational science

## Abstract

Geometry scaling of microwave circuits is an essential but challenging task. In particular, the employment of a given passive structure in a different application area often requires re-adjustment of the operating frequencies/bands while maintaining top performance. Achieving this necessitates the utilization of numerical optimization methods. Nonetheless, if the intended frequencies are distant from the ones at the starting point, local search procedures tend to fail, whereas global search algorithms are computationally expensive. As recently demonstrated, a combination of large-scale concurrent geometry parameter scaling with intermittent local tuning allows for dependable re-design of high-frequency circuits at low CPU costs. Unfortunately, the procedure is only applicable to single-band structures due to synchronized modifications of all operating bands under scaling. This article discusses a novel procedure that leverages a similar overall concept, but allows for independent control of all center frequencies. To achieve this goal, an automated decision-making procedure is developed in which a set of orthogonal scaling directions are determined based on their effect on individual circuit bands, and using auxiliary optimization sub-problems. The scaling range is then automatically computed by solving an appropriately-defined least-square design relocation problem. The methodology introduced in the work is illustrated using two planar passive devices. In both cases, wide-range operating frequency re-design has been demonstrated and favorably compared to conventional gradient-based tuning. Furthermore, the presented procedure has been shown to be computationally efficient. It is also easy to implement and integrate with a variety of gradient-based optimization procedures of a descent type.

## Introduction

Development of passive circuits relies on computational tools, such as electromagnetic (EM) analysis. Traditional methods involving analytical^1^ or equivalent network models^2^ are still used but reliable system evaluation requires EM analysis to account for dielectric and radiation losses, and cross-coupling effects^3^. As EM- and equivalent-network-evaluated responses differ, tuning of geometry parameters is imperative to boost the system performance^4,5^. In many cases, the bounded footprint and the multiband operation requirements, increase the complexity of the passives devices^6^. Handling of multiple objectives requires formal optimization procedures^7,8^. Yet, microwave design automation is a non-trivial matter. The difficulty is an excessive cost of repetitive EM analyses^9^. Conventional algorithms exhibit poor efficiency, which is pertinent even to local methods^10,11^, but dramatically pronounced for global procedures, especially nature-inspired routines^12–15^. Miniaturized devices constitute representative examples. On the one hand, popular size-reduction techniques (incorporation of slow-wave phenomenon^16^, compact microwave resonant cells, CMRCs^17^, transmission line meandering^18–20^, and many others^21–27^, lead to the enlargement of the parameter space dimensionality. On the other hand, non-intuitive relations between design variables and electrical characteristics exacerbate EM-driven design processes, especially optimization. In extreme cases, globalized search procedures^5,28, 29^ have to be employed, often resulting in extraordinary CPU expenses.

Expediting EM-driven design methodologies has been at the forefront of microwave CAD developments since late 1990s and early 2000s. In terms of local search algorithms, available methods include incorporation of adjoint sensitivities^30,31^ or mesh deformation^32^ for fast gradient evaluation, parallel computing^33^, and restricted Jacobian updating techniques^34–36^. In a more generic context, the popularity of surrogate-based optimization (SBO) approaches has increased immensely both for microwave^37–40^ and antenna design^41–44^. These include physics-based^45–47^, and behavioural modelling approaches (kriging^48^, neural networks^49^, support vector regression^50^, ensemble learning^51^, radial basis functions^52^), also in a variable-resolution regime (data blending by means of co-kriging^53^, two-stage Gaussian process regression^54^). Applicability of SBO methods is widespread and includes local^55^ and global parameter tuning^56^, multi-objective optimization^57^, as well as uncertainty quantification^58,59^. Among other techniques, one can mention response feature methods^60^, cognition-driven design^61^, along with machine learning (ML)^62,63^, many of which capitalize on the efficient global optimization (EGO) concept^64^. EGO involves iterative correction-prediction schemes interleaved with the allocation of infill samples and EM data acquisition to enhance the underlying surrogate model^65^. Some of the available techniques (frequency-based regularization^66^, adaptive design specifications^67^, parameter space pre-screening^68^) specifically target the search process reliability enhancements.

One of the optimization tasks that is particularly challenging yet important is geometry scaling of passive circuits to operating parameters of choice, which may be new center frequencies, but also material parameters (e.g., to implement the circuit on a different laminate). The difficulties linked to structure re-design include considerable computational expenses (as for any EM-driven procedure), but also the need to ensure reliability. It should be emphasized that if the intended center frequencies are significantly misaligned with the ones at the available design, traditional local tuning methods are likely to fail. At the same time, the employment of global algorithms is hindered by their poor computational efficiency. The methods developed to address these issues include analytical design curves^69^, response feature technology^70^, inverse surrogate modelling^71,72^, as well as advanced frameworks that permit independent control of the performance figures other than operating frequencies^73^. The primary disadvantage of the discussed approaches is high starting cost: in most cases, setting up the surrogate (whether forward or inverse) requires reference designs acquired beforehand, often through auxiliary optimization processes^71^. A potential alternative is utilization of general-purpose modelling methods^74–76^ with the metamodel rendered over a sufficiently large portion of the search space. However, this option only works for low-complexity circuits; for more sophisticated structures, building dependable surrogates is prevented by dimensionality-related issues. These and difficulties associated with broad ranges of parameters can be alleviated using recent performance-driven modelling techniques^77–79^, although even in this case, the initial setup cost may be significant^77^.

Recently, an attempt has been made to facilitate scaling of microwave passives by combining simultaneous geometry adjustment and local tuning^80^. This enables significant relocation of the center frequency/bandwidths in a reliable manner at a low computational cost. Unfortunately, the technique of^80^ is only applicable to single-band structures because the major scaling step therein is a concurrent scaling of all design variables, which normally leads to a synchronized change (up or down in frequency) of all operating frequencies. In this work, we introduce a generalized methodology, developed to handle multi-band circuits. Its keystone is the identification of an orthogonal set of scaling directions, which are determined to independently affect individual center frequencies. The knowledge-based scaling process is conducted along these directions, and it is interleaved with gradient-based tuning stages to enhance the circuit’s electrical performance figures. The latter facilitates subsequent scaling steps, and allows for the final design calibration upon relocating the operating parameters close enough to the assumed targets. Our procedure has been validated using two passive circuits. In both cases, broad-range operating frequency relocation capability has been demonstrated along with the computational efficiency, which is comparable to baseline gradient-based optimization. Furthermore, conventional local tuning has been shown to consistently fail due to significant misalignment between the operating parameters at the starting point and their intended values. In addition to its computational efficacy, the presented re-design procedure is easy to implement, and it does not incur any initial setup costs. Further, it only contains a few control parameters, which are intuitive to determine and essentially problem-independent.

The technical contribution and originality of this work can be summarized as follows: (i) development of a cost-efficient technique for re-design of multi-band microwave passive components with respect to target operating parameters (especially center frequency), (ii) development of rigorous approach to achieve quasi-independent scaling of individual operating bands by means of orthogonal scaling directions, (iii) development of a procedure for identification of scaling directions based on their effects on the circuit response variability and enforcing orthogonality, (iv) development of an automated re-design framework and demonstrating its capabilities using two multi-band passive structures under challenging design scenarios, (v) demonstrating quasi-global search capability without incurring excessive computational expenses. To the best knowledge of the authors the literature does not offer any technique that would be similar to the one proposed in this work in terms of the underlying methodology and performance.

## Multi-band microwave circuit scaling by orthogonal directions

Here, we provide the details of the proposed microwave circuit re-design algorithm. The scaling task is stated in Section "Multi-band circuit optimization. problem formulation". Following that, Section "Orthogonal scaling of microwave circuit geometry" explains the idea behind scaling directions and their extraction procedure. Section "Local tuning" outlines the local tuning stage. The complete framework has been outlined in Section "Optimization procedure"; its operation is also illustrated through a pseudocode and a flow diagram.

### Multi-band circuit optimization. problem formulation

We begin by recalling the formulation of the microwave optimization task. Table 1 explains the symbols and the terminology used throughout. In particular, we denote by *N* the number of operating bands, with *F*_*o.k*_ being the *k*th target center frequency. The objective is to relocate the device’s center frequencies towards their intended values, as well as to improve the system performance w.r.t. the figures of interest *F*_*p.j*_, *j* = 1, …, *K*. The performance of the design corresponding to the parameter vector ***x*** is measured using a scalar metric *U*(***x***,***F***_*o*_,***F***_*p*_). Accordingly, the highest-performance design, denoted as x^*^, is identified as follows

1 $$x^{*} = U^{*} ({\varvec{F}}_{o} ,{\varvec{F}}_{p} ) = \arg \mathop {\min }\limits_{x \in X} U(x,{\varvec{F}}_{o} ,{\varvec{F}}_{p} )$$

**Table 1:** EM-driven microwave design optimization: terminology.

Name	Symbol	Comments
Geometry parameter vector	***x*** = [*x*_1_ … *x*_*n*_]^*T*^	Independent geometry parameters to be adjusted during the re-design process
Parameter space	*X* = [***l u***]	Parameter space is an interval determined by the lower bounds ***l*** = [*l*_1_ … *l*_*n*_]^*T*^ and upper bounds ***u*** = [*u*_1_ … *u*_*n*_]^*T*^ on antenna parameters, i.e., we have *l*_*k*_ ≤ *x*_*k*_ ≤ *u*_*k*_,* k* = 1, …, *n*
Circuit responses	*S*_*kj*_(***x***,*f*)	Scattering parameters (*k* and *j* stand for the circuit ports) with explicit dependence on the parameter vector ***x*** and frequency *f* indicated
Target vector of operating frequencies	***F***_*o*_ = [*F*_*o*.0_ *F*_*o*.1_ … *F*_*o.N*_]^*T*^	*F*_*o.k*_ denotes the *k*th target operating frequency
Target vector of performance parameters	***F***_*p*_ = [*F*_*p*.0_ *F*_*p*.1_ … *F*_*p.K*_]^*T*^	*F*_*p.k*_ denotes the target value of the *k*th figure of interest (bandwidth, relative permittivity of the substrate, target power split ratio, etc.)
Objective function	*U*(***x***,***F***_*o*_,***F***_*p*_)	Scalar function quantifying the quality of design ***x*** w.r.t. the target vectors ***F***_*o*_ and ***F***_*p*_. It is defined so that better designs correspond to the lower values of *U*

In (1), *X* denotes the parameters space, which is normally an interval established using the respective ranges (lower ***l***, and upper ***u***, cf. Table 1). However, if required, additional constraints may be imposed, either pertaining to the circuit geometry (e.g., the maximum footprint area), or electrical characteristics. In this work, the latter are incorporated into the objective function, as discussed below.

Table 2 shows several illustrative examples of EM-driven design tasks along with the corresponding objective functions. In all cases, one of the objectives is handled directly (as the primary goal), whereas others are controlled using a penalty function approach^81^. The implicit approach is generally more convenient. Its primary advantage is that the design task becomes an unconstrained one^81^. This is particularly important in the case of expensive constraints, i.e., all that require EM analysis for their evaluation.

**Table 2:** Selected simulation-based design tasks for multi-band microwave components.

Task description	Target operating vector	Objective function^$^
Improve matching |*S*_11_| of impedance transformer at target frequencies *f*_0.*j*_, *j* = 1, …, *N*	***F***_*o*_ = [*F*_*o*.1_ … *F*_*o.N*_]^*T*^ ***F***_*p*_ = [ ]^*T*^ where *F*_*o.k*_ = *f*_*o.k*_—*k*th center frequency	$$U({\boldsymbol{x}},{\boldsymbol{F}}_{o} ,{\boldsymbol{F}}_{p} ) = \mathop {\max }\limits_{{k \in \{ 1,...,N\} }} |S_{11} ({\boldsymbol{x}},{\varvec{F}}_{o.k} )|$$
Improve matching |*S*_11_| and isolation |*S*_41_| of a microwave coupler, and ensure power split *d*_*S*_(***x***,*f*) =| |*S*_21_(***x***,*f*)|—|*S*_31_(***x***,*f*)| |= *K*_*j*_, both at target frequencies *f*_0.*j*_, *j* = 1, …, *N*.; the circuit is to be implemented on the substrate of permittivity ε_*r*_	***F***_*o*_ = [*F*_*o*.1_ … *F*_*o.N*_]^*T*^ ***F***_*p*_ = [*F*_*p*.1_ … *F*_*p.N*_* F*_*p.N*+1_]^*T*^ where *F*_*o.k*_ = *f*_*o.k*_—*k*th center frequency *F*_*p.k*_ = *K*_*k*_—*k*th target power split ratio *F*_*p.N*+1_ = ε_*r*_—substrate permittivity	$$\begin{gathered} U({\boldsymbol{x}},{\boldsymbol{F}}_{o} ,{\boldsymbol{F}}_{p} ) = \mathop {\max }\limits_{{k \in \{ 1,...,N\} }} \left\{ {|S_{11} ({\boldsymbol{x}},{\varvec{F}}_{o.k} )|,|S_{41} ({\boldsymbol{x}},{\varvec{F}}_{o.k} )|} \right\} \\ + \beta c({\boldsymbol{x}},{\boldsymbol{F}}_{o} ,{\boldsymbol{F}}_{p} )^{2} \\ \end{gathered}$$ where $$c({\boldsymbol{x}},{\boldsymbol{F}}_{o} ,{\boldsymbol{F}}_{p} ) = \left\| {\left[ \begin{gathered} |S_{31} ({\boldsymbol{x}},{\varvec{F}}_{o.1} )| - |S_{21} ({\boldsymbol{x}},{\varvec{F}}_{o.1} )| \\ \vdots \\ |S_{31} ({\mathbf{x}},{\varvec{F}}_{o.N} )| - |S_{21} ({\boldsymbol{x}},{\varvec{F}}_{o.N} )| \\ \end{gathered} \right] - \left[ \begin{gathered} {\varvec{F}}_{p.1} \\ \vdots \\ {\varvec{F}}_{p.N} \\ \end{gathered} \right]} \right\|$$
Reduce footprint *A*(***x***) of a microstrip coupler while maintaining matching and isolation at –20 dB or better, and equal power split ratio, both over operating bands *B*_*k*_, centered at *f*_*o.k*_, *k* = 1, …, *N*	***F***_*o*_ = [*F*_*o*.1_ … *F*_*o.N*_]^*T*^ ***F***_*p*_ = [*F*_*p*.1_ … *F*_*p.N*_* F*_*p.N*+1_]^*T*^ where *F*_*o.k*_ = *f*_*o.k*_—*k*th center frequency *F*_*p.k*_ = *B*_*k*_—*k*th operating bandwidth *F*_*p.N*+1_ = –20 dB—acceptance threshold for |*S*_11_| and |*S*_41_|	$$U({\boldsymbol{x}},{\boldsymbol{F}}_{o} ,{\boldsymbol{F}}_{p} ) = A({\boldsymbol{x}}) + \beta_{1} c_{1} ({\boldsymbol{x}},{\boldsymbol{F}}_{o} ,{\boldsymbol{F}}_{p} )^{2} + \beta_{2} c_{2} ({\boldsymbol{x}},{\boldsymbol{F}}_{o} ,{\boldsymbol{F}}_{p} )^{2}$$ where $$c_{1} ({\boldsymbol{x}},{\boldsymbol{F}}_{o} ,{\boldsymbol{F}}_{p} ) = \mathop {\max }\limits_{{k \in \{ 1,...,N\} }} \left\{ \begin{gathered} {\varvec{F}}_{o.k} - \frac{{{\varvec{F}}_{p.k} }}{2} \le f \le {\varvec{F}}_{o.k} - \frac{{{\varvec{F}}_{p.k} }}{2}: \\ \max \left\{ {\frac{{\max \left\{ {|S_{11} ({\boldsymbol{x}},f)|,|S_{41} ({\boldsymbol{x}},f)|} \right\} + {\varvec{F}}_{p.N + 1} }}{{{\varvec{F}}_{p.N + 1} }}} \right\} \\ \end{gathered} \right\}$$ $$c_{2} ({\boldsymbol{x}},{\boldsymbol{F}}_{o} ,{\boldsymbol{F}}_{p} ) = \mathop {\max }\limits_{{k \in \{ 1,...,N\} }} \left\{ {\left| {|S_{31} ({\boldsymbol{x}},{\varvec{F}}_{o.k} )| - |S_{21} ({\boldsymbol{x}},{\varvec{F}}_{o.k} )|} \right|} \right\}$$

^$^The coefficient *β* > 0 is a penalty factor controlling the contribution of the penalty terms to the objective function ^81^.

### Orthogonal scaling of microwave circuit geometry

Operating frequencies of conventional microstrip microwave components (e.g., transmission-line, TL-based) are well correlated with their physical dimensions^1,2^. Consequently, concurrent dimensions scaling normally leads to re-design of a circuit towards lower or higher center frequencies, depending on whether the structure is enlarged or reduced in size. On the other hand, the correlation is only approximate for more complex structures, especially miniaturized devices involving CMRCs^17^, metamaterial-based components^82^, defected ground structures^21^, etc. As a result, large-scale parameter adjustment is normally detrimental to electrical performance figures. Yet, the major issue in the context of multi-band circuits is that concurrent parameter adjustment normally affects all operating frequencies and bands in a similar way, i.e., leads to their simultaneous increase or decrease. The objective of this section is to introduce a technique for independent handling of individual center frequencies that retains the benefits of large-scale geometry adjustments. The presented methodology is rooted in the concept of orthogonal scaling directions as explained below based on the problem-specific knowledge.

#### Orthogonal scaling directions

**Figure 1:**
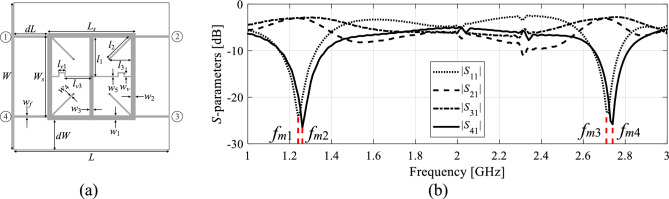
Extracting approximate center frequencies *f*_*a*.*k*_ of a coupler circuit: (**a**) coupler geometry, (**b**) *f*_*a*.1_ = (*f*_*m*1_ + *f*_*m*2_)/2, where *f*_*m*1_ and *f*_*m*2_ are the frequencies associated with the minima of |*S*_11_| and |*S*_41_| at the first operating band, and *f*_*a*.2_ = (*f*_*m*3_ + *f*_*m*4_)/2, where *f*_*m*3_ and *f*_*m*4_ are the frequencies associated with the minima of |*S*_11_| and |*S*_41_| at the second operating band.

We will use the notation *f*_0.*j*_(***x***) to mark the *j*th center frequency of the device associated with design ***x***, *j* = 1, …, *N*. The frequency is approximated using the EM-simulated *S*-parameters, e.g., by considering the local minima of respective responses (e.g., *S*_11_ and/or *S*_41_ for the coupling structures), cf. Figure 1. Identification of the operating frequencies might be tricky at times. In practice, the reflection response |*S*_11_| typically gives the best indication of the operating frequency, and therefore it is used for initial estimation of the operating conditions. The minima of other relevant responses (e.g., |*S*_41_| for a coupler) are then found in the vicinity of the previously found reflection minima. The aggregated vector of operating frequencies is marked using the symbol ***F***_*a*_(***x***) = [*f*_0.1_(***x***) … *f*_0.*N*_(***x***)]^*T*^. Here, the subscript ‘a’ stands for ‘aggregated’.

Below, we describe an automated procedure for determining a set of scaling vectors ***v***_*k*_, *k* = 1, …, *N*, such that altering the circuit geometry along ***v***_*k*_ only affects the center frequency *f*_0.*k*_, and has a possibly minimum effect on the remaining frequencies, *f*_0.*j*_, *j* ≠ *k*. Identification of the scaling directions requires repetitive evaluation of the circuit responses, therefore, it is executed using a model ***L***_*f*_^(*i*)^(***x***) approximating ***F***_*a*_(***x***). It is employed instead of EM analysis when generating a new candidate solution. The model ***L***_*f*_^(*i*)^(***x***) is determined at ***x***^(*i*)^ as

2 $${\varvec{L}}_{f}^{(i)} (x) = {\varvec{F}}_{a} (x^{(i)} ) + {\varvec{J}}_{F} (x^{(i)} ) \cdot (x - x^{(i)} )$$

In (2), the sensitivity matrix ***J***_*F*_(***x***) of ***F***_*a*_(***x***) is

3 $${\varvec{J}}_{F} (x) = \left[ {\begin{array}{*{20}c} {\frac{{\partial f_{0.1} (x)}}{{\partial x_{1} }}} & \cdots & {\frac{{\partial f_{0.1} (x)}}{{\partial x_{n} }}} \\ \vdots & \ddots & \vdots \\ {\frac{{\partial f_{0.N} (x)}}{{\partial x_{1} }}} & \cdots & {\frac{{\partial f_{0.N} (x)}}{{\partial x_{n} }}} \\ \end{array} } \right]$$

The Jacobian is estimated using finite differentiation; operating frequencies can be found by post-processing the circuit characteristics (cf. Figure 1). It is important to acknowledge that when evaluating circuit responses through electromagnetic (EM) analysis, some degree of numerical noise is inevitable. Factors such as adaptive meshing within the solver and termination criteria of the simulation process contribute to this noise. Consequently, finite differentiation steps need to be chosen significantly larger than those typically used for analytical objective functions. To address this, we start with a perturbation step of 0.1 mm and then adjust it to ensure that the change in response is noticeable (as determined through visual inspection) but not excessive. In practice, the range of parameter perturbations typically falls between 0.02 and 0.2 mm in most cases. On the other hand, it should be reiterated that the dependence of the operating parameters on design variables is much more regular than a similar dependence for the complete frequency characteristics, which make the Jacobian in (3) less prone to inaccuracy^83^.

The scaling directions ***v***_*k*_ are found by maximizing the operating frequency variability functionals Δ*F*_*k*_, *k* = 1, …, *N*, defined as

4 $$\Delta {\varvec{F}}_{k} \left( {{\varvec{F}}_{1} ,{\varvec{F}}_{2} } \right) = \Delta {\varvec{F}}_{k} \left( {\left[ {{\varvec{F}}_{1.1} \;...\;{\varvec{F}}_{1.N} } \right]^{T} ,\left[ {{\varvec{F}}_{2.1} \;...\;{\varvec{F}}_{2.N} } \right]^{T} } \right) = \left( {{\varvec{F}}_{1.k} - {\varvec{F}}_{2.k} } \right)^{2} - \alpha \sum\limits_{\substack{ j = 1,...,N \\ j \ne k } }^{{}} {\left( {{\varvec{F}}_{1.j} - {\varvec{F}}_{2.j} } \right)^{2} }$$

It can be observed that Δ*F*_*k*_ consists of two components. The first one is a measure of *k*th operating frequency relocation to be maximized, whereas the second is a regularization term introduced to enforce a possibly minimum relocation of the remaining operating frequencies. Note that Δ*F*_*k*_ is a smooth function of ***x*** when evaluated using ***L***_*f*_^(*i*)^. Consequently, the proportionality coefficient can be set to relatively high values, e.g., *α* = 1,000, without leading to numerical issues.

The vector ***v***_1_ is found by unconstrained maximization of the functional Δ*F*_1_. We have

5 $$v_{1} = \arg \mathop {\max }\limits_{v;\;||v|| = 1} \Delta {\varvec{F}}_{1} \left( {{\varvec{L}}_{f}^{(i)} \left( {x^{(i)} + v} \right),{\varvec{F}}_{a} \left( {x^{(i)} } \right)} \right)$$

Identification of the remaining vectors is carried out by taking into account orthogonality conditions. In particular, given ***v***_*j*_, *j* = 1, …, *k*, the subsequent vector ***v***_*k*+1_ is found as

6 $$v_{k + 1} = \arg \mathop {\max }\limits_{{\overline{v}}} \Delta {\varvec{F}}_{k + 1} \left( {{\varvec{L}}_{f}^{(i)} \left( {x^{(i)} + v} \right),\;{\varvec{F}}_{a} \left( {x^{(i)} } \right)} \right)$$

where

7 $$\overline{v} = \frac{{P^{(k)} (v)}}{{||P^{(k)} (v)||}}$$

in which the orthogonal projection *P*^(*k*)^(***v***) of ***v*** onto the linear subspace spanned by ***v***_*j*_, *j* = 1, …, *k*, is defined as

8 $$P^{(k)} (v) = v - \sum\nolimits_{j = 1}^{k} {v_{j} \left[ {(v_{j} )^{T} v} \right]}$$

Note that the process (6–8) guarantees that ***v***_*j*_ ⊥ ***v***_*k*_ for *j* ≠ *k*, and ||***v***_*k*_||= 1 for *k* = 1, …, *N*.

#### Geometry scaling algorithm

Geometry scaling aims at relocating the center frequencies of the circuit (vector ***F***_*a*_(***x***)) towards the target ***F***_*o*_. The scaling is realized along the directions {***v***_*k*_}_*k* = 1, …, *N*_, identified in Section "Orthogonal scaling directions". The first step is to estimate the large-scale sensitivity of center frequencies to parameter adjustments along vectors ***v***_*k*_. To this end, we take a positive number *d* (e.g., *d* = 0.1), and, given ***x***^(*i*)^, assign the perturbed vectors

9 $$x_{d.k} = x^{(i)} + dv_{k} , \;\;k = \, 1, \, \ldots ,N$$

The EM-simulated circuit characteristics corresponding to ***x***_*d.k*_ are used to extract the corresponding center frequencies ***F***_*a*_(***x***_*d.k*_). The operating frequency sensitivities are estimated as

10 $$\nabla {\varvec{F}}_{a.k} (x^{(i)} ) = \frac{{{\varvec{F}}_{a} (x_{d.k} ) - F(x^{(i)} )}}{d} = \frac{{{\varvec{F}}_{a} (x^{(i)} + dv_{k} ) - F(x^{(i)} )}}{d}, \;\;k = \, 1, \, \ldots ,N$$

The gradients ∇***F***_*a.k*_(***x***^(*i*)^) are then employed to determine the range of geometry scaling. More specifically, consider the equation

11 $${\varvec{F}}_{a} (x^{(i)} ) + {\varvec{J}}_{F} (x^{(i)} )h = {\varvec{F}}_{o}$$

where ***h*** is the unknown (vector-valued) scaling step, and

12 $${\varvec{J}}_{F} (x^{(i)} ) = \left[ {\nabla {\varvec{F}}_{a.1} (x^{(i)} )\;\;...\;\;\nabla {\varvec{F}}_{a.N} (x^{(i)} )} \right]$$

It can be observed that the *N* × *N* Jacobian matrix ***J***_***F***_ is non-singular. More specifically, as the directions ***v***_*k*_ have been established to mainly affect the center frequency *f*_0.*k*_, thus ***J***_***F***_ is diagonally dominant, therefore invertible. This property leads to an analytical solution of (11), which is of the form

13 $$h = \left[ {{\varvec{J}}_{F} (x^{(i)} )} \right]^{ - 1} \left( {{\varvec{F}}_{o} - {\varvec{F}}_{a} (x^{(i)} )} \right)$$

The re-located design is

14 $$x_{scaled} = x^{(i)} + Vh$$

where

15 $$V = \left[ {\begin{array}{*{20}c} {v_{1} } & \cdots & {v_{N} } \\ \end{array} } \right]$$

It is important to mention that although analytical solution (13) is convenient to use, it may not be appropriate at times as it does not give sufficient control over the location of the vector ***x***_*scaled*_. In particular, it may happen that ***x***_*scaled*_ is outside the domain *X*, which would require ‘trimming’ it to fit within the original parameter space [***l***, ***u***]. If any additional geometry constraints are in place, utilization of (13) becomes even more problematic. In order to mitigate these potential issues, here, an optimization-driven geometry scaling is applied, in which the scaling step is obtained as

16 $$h = \arg \mathop {\min }\limits_{{\overline{h}}} \left\| {{\varvec{F}}_{a} (x^{(i)} ) + {\varvec{J}}_{F} (x^{(i)} )\overline{h} - {\varvec{F}}_{o} } \right\|$$

subject to

17 $$x^{(i)} + V\overline{h} \in X$$

The problem (16) is, in fact, a nonlinear least-square task. Upon finding ***h***, the design ***x***_*scaled*_ of (14) is used to replace ***x***^(*i*)^ during optimization.

Regardless of the approach (analytical (13) or optimization-driven (16), (17)), the fact that the scaling directions may not be exclusively affecting the prescribed center frequencies (i.e., vector ***v***_*k*_ may also alter frequencies *f*_0.*j*_, *j* ≠ *k*, to a certain extent), is accounted for. To conclude this, observe that the system (11) corresponds to a linear combination of the gradients ∇***F***_*a.k*_(***x***^(*i*)^), which can span the difference vector ***F***_*a*_(***x***^(*i*)^)—***F***_*o*_ due to the fact that vectors {∇***F***_*a.k*_(***x***^(*i*)^)}_*k* = 1, …, *N*_ are linearly independent in the operating frequency space, therefore, they form a basis therein.

In some cases, the parameter space may be quite large in terms of the parameter ranges ***u***—***l***. If, additionally, the device’s center frequencies at the starting point ***F***_*a*_(***x***^(0)^) are misaligned with ***F***_*o*_, extensive design relocation might be expected due to geometry scaling, which results in deteriorating performance. In such situations, a more advantageous approach would be to limit the extent of scaling to a smaller neighborhood of the current design by imposing a constraint.

18 $$x^{\left( i \right)} {-}M\left( {u{-}l} \right)\, \le \,x^{(i)} \, + \,Vh\, \le \,x^{(i)} \, + \,M\left( {u{-}l} \right)$$

The inequalities in (18) are component-wise; 0 < *M* < 1 is a control parameter of the procedure.

**Figure 2:**
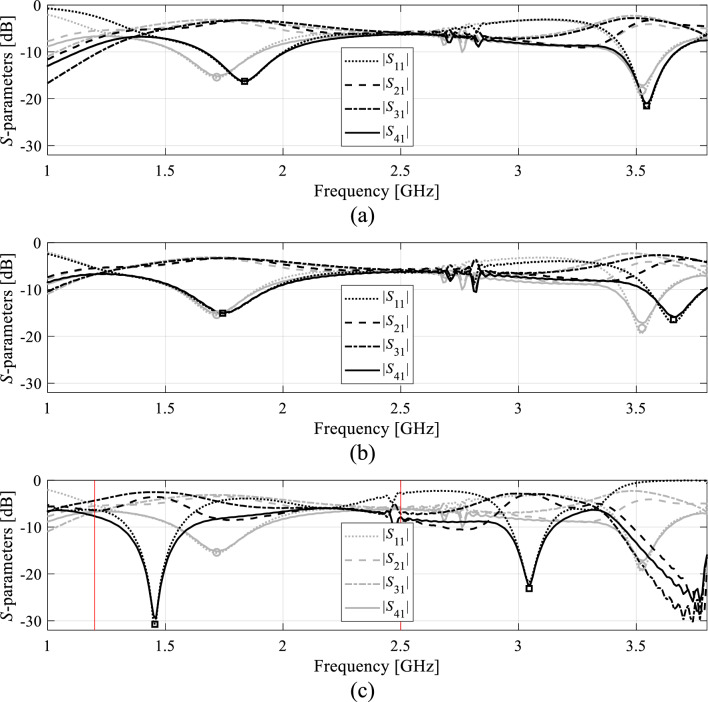
Orthogonal scaling directions for a dual-band branch-line coupler of Fig. 1(a): (**a**) the effects of the scaling vector ***v***_1_ assuming an example step size *h* = 0.5 (grey—initial design, black—perturbed design); (**b**) the effects of the scaling vector ***v***_2_ assuming an example step size *h* = 0.5 (grey—initial design, black—perturbed design); (**c**) the effects of concurrent geometry scaling (13)-(15) using both directions and example target frequencies *F*_*o.*1_ = 1.2 GHz and *F*_*o*.2_ = 2.5 GHz (grey—initial design, black—scaled design). Considerable relocation of both center frequencies towards the target can be observed.

Figure 2 illustrates the operation of the automated orthogonal geometry scaling using the coupler shown in Fig. 1(a). Figure 2(a) and (b) demonstrate the effects of moving along the vectors ***v***_1_ and ***v***_2_; it is evident that both vectors allow for controlling the lower and upper center frequency almost independently. Figure 2(c) shows the effects of concurrent scaling according to (13)–(15), assuming the target vector ***F***_*o*_ = [1.2 2.5]^*T*^ GHz. A significant center frequency relocation towards the target can be observed. Achieving perfect alignment between ***F***_*a*_ and ***F***_*o*_ normally requires several iterations (cf. Section "Algorithm validation").

The procedure is computationally efficient. Each scaling stage only needs *n* + *N* full-wave analyzes of the structure (*n* and *N* represent the number of geometry parameters and operating bands, resp.). Note that these expenses are in line with the cost of a single iteration of gradient-based optimizer (given that the Jacobian is evaluated using finite differentiation), therefore, the overall re-design cost is higher by a multiplicative factor of roughly two over the cost of local tuning at the early stages thereof. Upon aligning ***F***_*a*_ and ***F***_*o*_, concurrent scaling is not executed anymore. The mentioned additional expenses are minor given that the proposed procedure enables quasi-global search capabilities. The computational efforts associated with global (e.g., nature-inspired) algorithms are significantly higher.

### Local tuning

Geometry scaling described in Section "Orthogonal scaling of microwave circuit geometry" addresses the main problem related to large-scale circuit re-design, which is the relocation of the operating frequencies. However, scaling does not provide any means to control the performance figures of the circuit such as power split ratio or matching/isolation characteristics. For that, we need a supplementary tool, which is local parameter tuning. Local parameter adjustment is applied between the geometry scaling stages. Its role is performance improvement, that is, reducing the merit function *U*(***x***,***F***_*a*_,***F***_*p*_), as a preparation for further scaling steps. This is essential as extensive scaling results in a deterioration of the circuit responses. Furthermore, after the center frequencies ***F***_*a*_ have been aligned with the target vector ***F***_*o*_, only local tuning is employed in the remaining part of the geometry scaling procedure.

Local tuning is carried out by means of the the trust-region routine^84^. The structure’s Jacobian matrix is computed through finite differentiation (FD)^85^. The candidate parameter vector ***x***^(*i*+1)^ is sought for in the neighbourhood of ***x***^(*i*)^ (current iteration point) as

19 $$x^{(i + 1)} = \arg \mathop {\min }\limits_{\substack{ x \in X \\ ||x - x^{(i)} || < r^{(i)} } } U_{L} \left( {x,{\varvec{F}}_{a} ,{\varvec{F}}_{p} } \right)$$

The analytical form of *U*_*L*_ is identical to *U* (cf. Section "Multi-band circuit optimization. problem formulation"). However, *U*_*L*_ is evaluated using the approximation model

20 $$L^{(i)} \left( x \right) = S\left( {x^{(i)} } \right) + {\varvec{J}}_{S} \left( {x^{(i)} } \right) \cdot \left( {x - x^{(i)} } \right)$$

As mentioned earlier, the sensitivity matrix ***J***_*S*_ is estimated by FD ^85^. It should also be emphasized that problem (19) is formulated w.r.t. the current vector ***F***_*a*_(***x***^(*i*)^), not with respect to ***F***_*o*_ as in the original task (1). This is to improve the performance figures exactly at ***F***_*a*_, thereby having the design ***x***^(*i*+1)^ better prepared to the next geometry scaling stage.

The size parameter *r*^(*i*)^ in (19) is altered after each iteration using the so-called gain ratio

21 $$\rho = \frac{{U\left( {x^{(i + 1)} ,{\varvec{F}}_{a} ,{\varvec{F}}_{p} } \right) - U\left( {x^{(i)} ,{\varvec{F}}_{a} ,{\varvec{F}}_{p} } \right)}}{{U_{L} \left( {x^{(i + 1)} ,{\varvec{F}}_{a} ,{\varvec{F}}_{p} } \right) - U_{L} \left( {x^{(i)} ,{\varvec{F}}_{a} ,{\varvec{F}}_{p} } \right)}}$$

Note that *ρ* compares the actual (i.e., EM-evaluated) improvement of the merit function *U* to the improvement predicted using the linear model ***L***^(*i*)^. To accept the design ***x***^(*i*+1)^ it is necessary that *ρ* > 0. If *ρ* ≤ 0, the iteration needs to be redone using a diminished *r*^(*i*)^. The typical updating rules work as follows: if *ρ* > 0.75 then *r*^(*i*+1)^ = 2*r*^(*i*)^, and if *ρ* < 0.25 then *r*^(*i*+1)^ = *r*^(*i*)^/3; otherwise the radius stay intact^84^.

If the circuit of interest can be designed for the operating frequencies ***F***_*o*_, i.e., finding the parameter vector ***x*** for which ***F***_*a*_(***x***) = ***F***_*o*_ is possible, it usually requires a few iterations of the geometry scaling combined with local adjustment (19), (20) to achieve good operating frequency alignment. Subsequently, the gradient-based algorithm remains the only optimization procedure. It is then executed until convergence. In this work, we use the following termination conditions:

22 $$\left| {\left| {\boldsymbol{x}^{(i + 1)} {-}\boldsymbol{x}^{(i)} } \right|} \right| \, < e_{x} \;\;{\text{OR }}\;\; r^{(i)} < e_{x}$$

corresponding to convergence in argument and sufficient search radius reduction, respectively. The resolution of the search process is decided upon by the designer (typically, *ε*_*x*_ = 10^–3^). The computational cost of the tuning process is approximately O(*n*) per iteration, and it is also a complexity of the prior steps (scaling), with the cost associated with numerical evaluation of the circuit response gradients.

### Optimization procedure

Sections "Orthogonal scaling of microwave circuit geometry" and "Local tuning" introduced the two major components of the considered methodology, orthogonal geometry scaling, and final parameter adjustment. The procedure is initiated with the scaling step, therefore, it is assumed that the starting point ***x***^(0)^ is of sufficient quality as measured by *U*(***x***^(0)^,***F***_*a*_(***x***^(0)^),***F***_*p*_). This assumption is normally satisfied because the re-design process typically starts from the design that was optimized before with respect to another set of target operating frequencies. However, if the quality of ***x***^(0)^ is insufficient, local optimization should be executed before launching the procedure introduced in this study.

The control parameters of the algorithm can be found in Table 3. Leaving alone the termination coefficient *ε*_*x*_, used to decide upon the search process resolution, we only have two parameters: *dF*_0_ and *M*. The former decides whether to execute the geometry scaling step, and should be maintained at the level of a fraction (e.g., 10%) of ||***F***_*p*_||. With this arrangement, satisfaction of the condition ||***F***_*a*_* – ****F***_*o*_||< *dF*_0_ essentially guarantees that target frequencies are attainable from the current design. The parameter *M* is normally set to unity; however, it may be reduced if the initial conditions of the re-design process are particularly challenging (e.g., large initial distance between ***F***_*a*_ and ***F***_*p*_).

**Table 3:** Summary of the algorithm’s control parameters.

Parameter	Default value	Explanations
*dF*_0_	0.05⋅||***F***_*o*_||	Orthogonal geometry scaling threshold (scaling enabled if ||***F***_*a*_ − ***F***_*o*_||> *dF*_0_)
*M*	1.0	Relative range of orthogonal geometry scaling (scaling limited to the interval [***x***^(*i*)^ − *M*(***u*** − ***l***), ***x***^(*i*)^ + *M*(***u*** − ***l***)])
*ε*_*x*_	10^–3^	Termination threshold (cf. Section "Local tuning")

**Figure 3:**
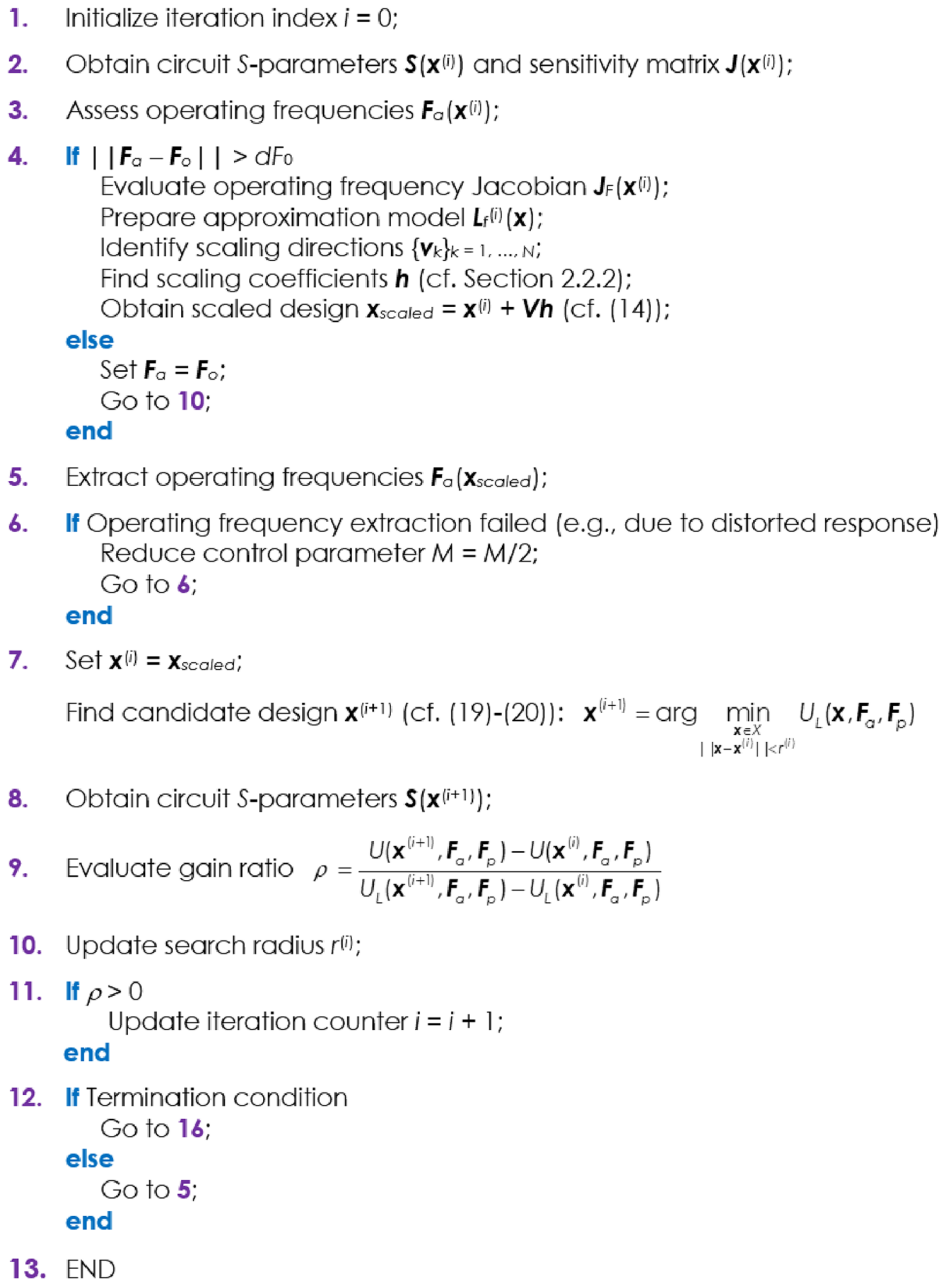
Operating principles of the considered technique for geometry scaling of multi-band microwave structures using orthogonal geometry scaling and local tuning. The following input arguments need to be supplied: ***x***^(0)^—initial design, ***F***_*o*_—target vector (operating frequencies), ***F***_*p*_—target vector (performance figures), *X*—parameter space, *U*—objective function;

The operating principles of the proposed procedure are summarized in Fig. 3. Note that the geometry scaling step is launched if the operating conditions at ***x***^(*i*)^ are away from their intended values, i.e., if ||***F***_*a* − _***F***_*o*_||> *dF*_0_. In the case of a failure (e.g., if the circuit responses at the scaled design are misshaped so that the vector ***F***_*a*_ cannot be extracted), the step is repeated with a reduced value of the parameter *M*.

The second essential part of the procedure is the gradient-based parameter adjustment (Step 10). Therein, we aim at reducing *U*(***x***,***F***_*a*_,***F***_*p*_) as much as possible, which facilitates further scaling steps. At the same time, if ||***F***_*a*_(***x***^(*i*)^)_ − _***F***_*o*_||< *dF*_0_ at ***x***^(*i*)^, scaling is no longer carried out, and local tuning becomes the only process, which is continued until convergence. At this stage, the function being minimized is *U*(***x***,***F***_*o*_,***F***_*p*_), i.e., the performance of the device is improved w.r.t. the original target vector ***F***_*o*_.

In terms of computational expenses, the procedure involves three main steps: (i) identifying scaling directions, (ii) applying them to scale the design, and (iii) correcting the design to enhance primary performance metrics. Importantly, none of these steps are significantly affected by the curse of dimensionality. Identifying scaling directions relies on the circuit response Jacobian, hence its computational costs are independent of the number of operating frequencies. Similarly, applying these directions to scale the design entails minimal computational overhead, regardless of the frequency count. Design correction, the third step, also relies on a linear expansion model, implying that overall costs of the redesign process mirror those of conventional trust-region gradient-based searches. These costs scale linearly with the parameter space's dimensionality. While increasing the number of operating frequencies may necessitate more algorithm iterations, the impact on computational expenses is marginal.

It is important to acknowledge that for certain circuits, there are limitations on independently tuning the operating frequencies within a specific range. This implies that it may not always be feasible to adjust them to arbitrarily selected targets. Consequently, if this restriction exists, it would be reflected in the scaling directions affecting two or more operating bands. It is essential to recognize that this limitation stems from the circuit itself rather than being a constraint of the proposed method.

## Algorithm validation

In this part of the article, we validate the scaling methodology discussed in Section "Multi-band microwave circuit scaling by orthogonal directions". Our numerical experiments involve two dual-band passive microstrip circuits. The geometry parameters of the circuits are re-adjusted to improve the alignment between their operating parameters and their assumed target values. Under these conditions, conventional local optimization is unable to identify satisfactory designs. In contrast to that, our approach is demonstrated to work successfully, especially in terms of appropriate manipulation of the center frequencies.

### Verification structures

**Figure 4:**
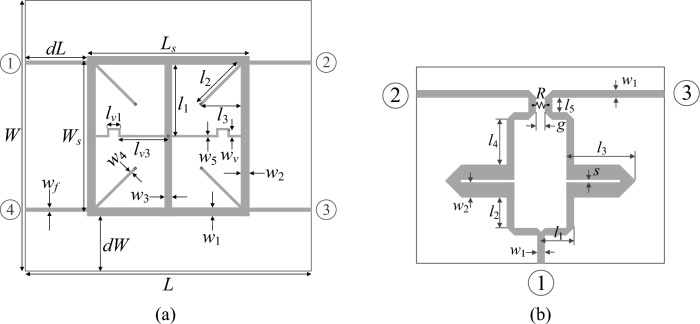
Microstrip circuits utilized as the test cases to demonstrated the geometry scaling algorithm introduced in this paper: (**a**) dual-band branch-line coupler (Circuit I)^86^, (**b**) dual-band power divider (Circuit II)^87^.

For the purpose of validation, we utilize two dual-band passive structures, a compact branch-line coupler (Circuit I), as well as a power divider (Circuit II). The circuit geometries have been shown in Fig. 4, whereas Table 4 gathers the basic parameters of the structures, e.g., relative permittivity and thickness of the substrate, adjustable geometry parameters, target center frequencies, initial designs, etc. The considered structures are evaluated in CST MWS; the simulation process is carried out with the time-domain solver. For both circuits, we aim at aligning their operating frequencies with those contained in the target vectors ***F***_*o*_ listed in Table 4, and to maintain equal power division. Furthermore, for Circuit I, we aim at minimizing |*S*_11_| as well as |*S*_41_| (port isolation) at the assumed operating frequencies. The objective function *U* is implemented as shown in Table 2 (the second row). It should be mentioned that the equal power split property of the circuit is enforced using a penalty term *c*. For Circuit II, we aim at a concurrent reduction of the following characteristics: |*S*_11_| (input impedance matching), |*S*_22_|, |*S*_33_| (output impedance matching), and |*S*_23_| (port isolation). The objective function is implemented similarly as for Circuit I; however, equal power division is achieved automatically due to the geometrical symmetry of the device.

**Table 4:** Verification circuits.

	Microwave structure	
	Circuit I	Circuit II
Substrate	RO4003 (*ε*_*r*_ = 3.5, *h* = 0.51 mm)	AD250 (*ε*_*r*_ = 2.5, *h* = 0.81 mm)
Design parameters	***x*** = [*L*_*s*_* W*_*s*_* l*_3*r*_* w*_1_ *w*_2_ *w*_3_ *w*_4_ *w*_5_ *w*_*v*_]^*T*^	***x*** = [*l*_1_* l*_2_* l*_3_* l*_4_* l*_5_ *s w*_2_]^*T*^
Other parameters	*d*_*L*_ = *d*_*W*_ = 10 mm, *L* = 2*d*_*L*_ + *L*_*s*_*, W* = 2*d*_*W*_ + 2*w*_1 _+ (*W*_*s*_ − 2*w*_*f*_),* l*_1_ = *W*_*s*_/2*, l*_2_ = *l*_3_2^1/2^,* l*_3_ = *l*_*3r*_((*L*_*s*_ − *w*_3_)/2 − *w*_*4*_/2^1/2^), *l*_*v*1_ = *l*_3_/3,* l*_*v*3_ = *L*_*s*_/2—*w*_3_/2 − *l*_3_ + *l*_*v*1_,* w*_*f*_ = 1.15 mm	*w*_1_ = 2.2 mm, *g* = 1 mm
Target operating frequencies	***F***_*o*_ = [1.2 2.5]^*T*^ GHz	***F***_*o*_ = [2.4 3.8]^*T*^ GHz
Target power division ratio	*K* = 0 dB	*K* = 0 dB
Parameter space	***l*** = [12 –10 0.3 0.5 0.5 0.3 0.3 0.3 0.1]^*T*^ ***u*** = [80 10 0.9 3.0 2.0 2.0 2.0 2.0 3.0]^*T*^	***l*** = [5.0 2.0 10.0 2.0 1.0 0.1 1.5]^*T*^ ***u*** = [40.0 20.0 50.0 15.0 6.0 0.5 8.0]^*T*^
Initial design	***x***^(0)^ = [35 0 0.9 1.6 1.0 0.75 0.6 0.4 0.5]^*T*^	***x***^(0)^ = [30.0 15.0 35.0 12.0 3.0 0.4 6.0]^*T*^
Operating frequencies at ***x***^(0)^	***F***_*a*_(***x***^(0)^) = [1.72 3.53]^*T*^ GHz	***F***_*a*_(***x***^(0)^) = [1.4 2.05]^*T*^ GHz

It should be emphasized that the actual center frequencies of our verification structures are away from the targets. Consequently, straightforward gradient-based parameter tuning is unlikely to yield satisfactory designs.

### Results

Circuits I and II underwent a re-design procedure using the algorithm proposed in Section "Multi-band microwave circuit scaling by orthogonal directions", assuming the target vectors ***F***_*o*_ shown in Table 4. Conventional gradient-based algorithm^84^ was also applied for the sake of comparison. As expected, significant discrepancy between ***F***_*a*_(***x***^(0)^) and ***F***_*o*_ led to a failure of local tuning in both cases. At the same time, the procedure of Section "Multi-band microwave circuit scaling by orthogonal directions" has been successful in bringing the center frequencies towards ***F***_*o*_.

**Figure 5:**
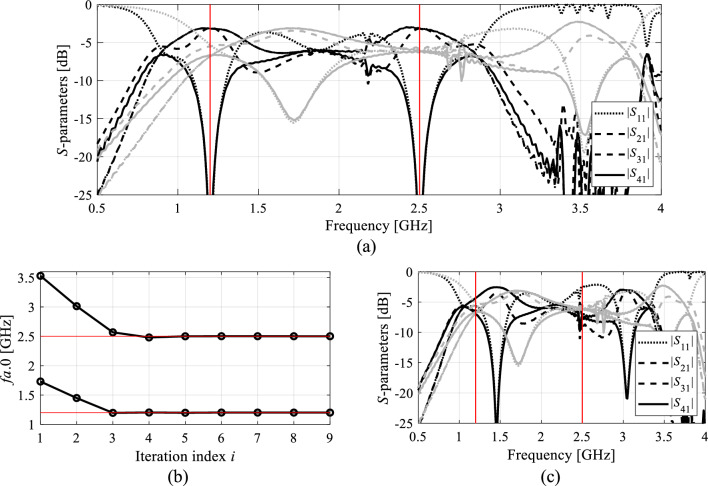
Circuit I: (**a**) EM-simulated characteristics at the initial (grey) and final design (black); (**b**) operating frequencies ***F***_*a*_ versus iteration index; (**c**) circuit responses after the first geometry scaling (black) compared to characteristics at the initial design (grey).

**Figure 6:**
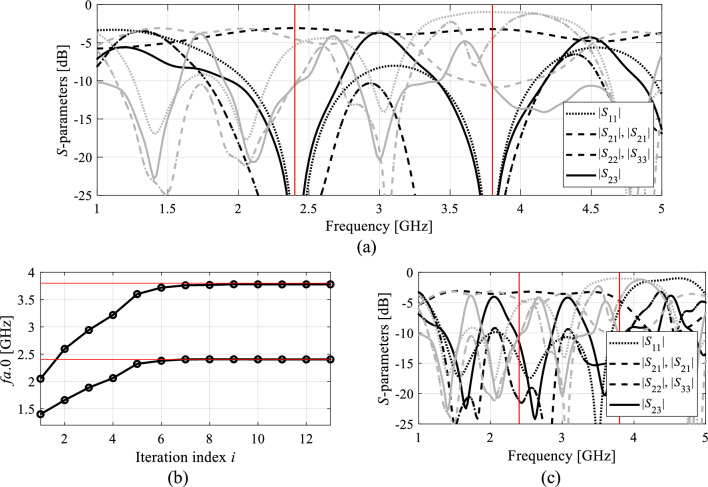
Circuit II: (**a**) EM-simulated characteristics at the initial (grey) and final design (black); (**b**) operating frequencies ***F***_*a*_ versus iteration index; (**c**) circuit responses after the first geometry scaling (black) compared to characteristics at the initial design (grey).

As shown in Table 5, the vectors ***F***_*a*_(x^*^) and ***F***_*o*_ coincide for Circuit I and II. Furthermore, the presented algorithm is capable of considerably improving the performance figures, in particular, achieving excellent impedance matching (both input and output), and port isolation levels at both operating frequencies, along with maintaining equal power split ratio. The optimized circuit characteristics can be found in Figs. 5 and 6.

**Table 5:** Summary of geometry scaling results for Circuits I and II.

Circuit	Target operating frequencies [GHz]	Actual operating frequencies [GHz]	Geometry parameter values
I	[1.2 2.5]	[1.2 2.5]	x* = [50.6 1.76 0.63 2.24 1.82 0.76 0.69 2.00 0.29]^*T*^
II	[2.4 3.8]	[2.4 3.8]	x* = [17.7 7.89 21.5 5.34 0.60 1.19 5.85]^*T*^

x* - optimal solution.

The same pictures also illustrate the history of the center frequencies of the circuits, as well as the response alterations upon accomplishing the first geometry scaling stage. As it turns out, perfect realignment of ***F***_*a*_(***x***) with ***F***_*o*_ requires several iterations of the optimization process. The process takes longer for Circuit II, because scaling is quite detrimental for its performance figures. Upon relocating the center frequencies to their target values, the remaining part of the optimization process is essentially local tuning that improves *U*(***x***,***F***_*o*_,***F***_*p*_). From the perspective of *U* (specifically, its second argument), the process of geometry scaling interleaved by local improvements can be viewed as an iterative adjustment of the design goals, guided by the current operating vector ***F***_*a*_.

The cost efficiency of the proposed geometry scaling algorithm is excellent given its quasi-global search capability. The CPU expenses correspond to 125 and 179 EM simulations for Circuit I and II, respectively, which is comparable to the cost of local optimization. It can be noted that large-scale alteration of the center frequencies normally requires resorting to global search methods, which is associated with considerably larger costs (typically from several hundreds to many thousands of full-wave simulations, especially in the case of nature-inspired algorithms).

Reliability, the capability of re-designing multi-band components over broad ranges of center frequencies, as well as computational efficiency, are all attractive features of the presented approach. Another one, important from a design utility standpoint is simple implementation along with a very limited number of control coefficients. Apart from the termination condition, which is a generic variable used to determine the search process resolution, we only have a single essential parameter *dF*_0_, which is problem-dependent. As mentioned before, to be on a safe side, it is sufficient to set it up to a small fraction of the typical operating bandwidth of the circuit at hand (a few percent thereof) in order to warrant reachability of the optimum design from the parameter vector satisfying ||***F***_*a*_(***x***)_ − _***F***_*o*_||< *dF*_0_.

**Figure 7:**
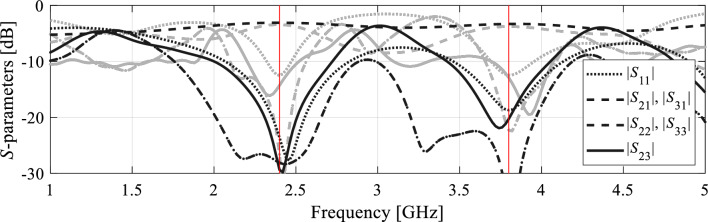
Optimization of Circuit I using the trust-region algorihm (black) and PSO (gray). Shown are EM-simulated circuit characteristics at the final designs produced by both algorithms. The target operating frequencies marked using vertical lines.

**Figure 8:**
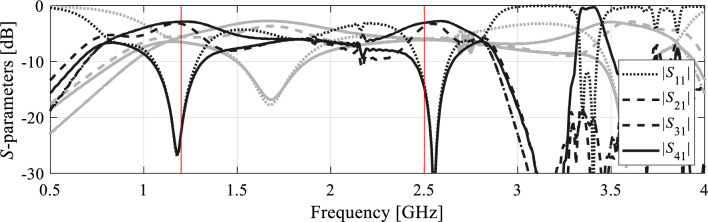
Optimization of Circuit II using the trust-region algorihm (black) and PSO (gray). Shown are EM-simulated circuit characteristics at the final designs produced by both algorithms. The target operating frequencies marked using vertical lines.

The proposed algorithm has been compared to local and global search procedures, specifically the trust-region algorithm^84^, and particle swarm optimizers (PSO)^88^ used as a representative nature-inspired method. Both algorithms exhibit poor performance. The optimization cost of the gradient-based algorithm is 195 and 108 for Circuit I and II, respectively. In neither case, the algorithm was capable of identifying a satisfactory design, as illustrated in Figs. 7 and 8. On the other hand, PSO was executed with the computational budget of 1000 EM simulations (swarm size of 10, maximum number of iterations 100). Yet, the obtained results are rather mediocre as shown in Figs. 7 and 8.

**Figure 9:**
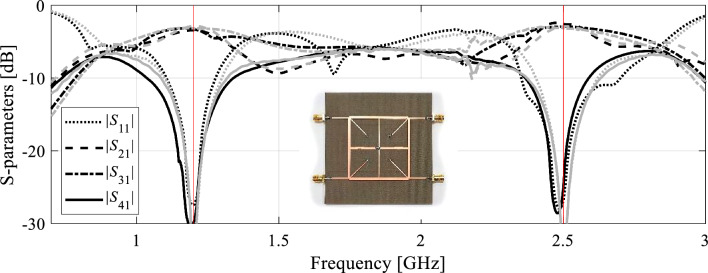
Experimental validation of Circuit I: EM-evaluated and experimentally acquired scattering parameters (marked grey and black, respectively). The inset shows a photograph of the fabricated circuit prototype.

**Figure 10:**
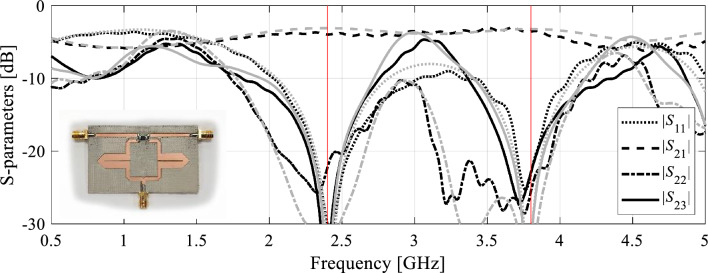
Experimental validation of Circuit II: EM-evaluated and experimentally acquired scattering parameters (marked grey and black, respectively). The inset shows a photograph of the fabricated circuit prototype.

For supplementary verification, the final designs of both test circuits yielded by means of the proposed technique have been fabricated and experimentally validated. Geometry parameters of the structures are set as listed in Table 4. Figures 9 and 10 show the photographs of the prototypes of Circuit I and II, respectively, along with their EM-simulated and measured scattering parameters. As it can be observed, the alignment between the simulations and experimentally acquired responses is excellent. Minor discrepancies might be attributed to manufacturing and assembly inaccuracies.

## Conclusion

In this study, we elaborated on an approach to large-scale frequency scaling of multi-band passive circuits. The presented methodology incorporates automated geometry scaling realized along a set of orthogonal directions, identified to affect individual center frequencies of the structure at hand, as well as local (gradient-based) tuning. The two steps are interleaved so that parameter scaling is followed by the improvement of electrical performance parameters, as a preparation for further dimension modifications. Upon relocating the center frequencies of the system close enough to their intended values, gradient-based optimization is continued until convergence. The major distinctive feature of the presented approach is to permit relatively large and low-CPU-expense adjustments of the system geometry parameters while ensuring sufficient design quality during the optimization run. Effectively, this enables a quasi-global search capability while incurring expenses similar to traditional local tuning algorithms.

The proposed technique has been applied to two dual-band passive circuits of distinct characteristics. The starting points were selected to be away from the respective optima with respect to the allocation of the operating bands. Under such conditions, conventional local optimization failed, whereas the presented framework demonstrated its efficacy in terms of dependability and low running costs. It is noteworthy that the perfect allocation of the center frequencies with the targets was achieved within a few iterations of the search process. The typical CPU expenses required to determine the final design correspond to just 150 EM analyses of the device under design.

The geometry scaling methodology presented in this study may be considered an attractive technique for dimension scaling of multi-band circuits, especially when the computational budget is of concern. Apart from its computational efficiency and reliability, it also exhibits practical advantages of being easy to handle due to only a handful of control parameters, which are straightforward to set up.

The future work will be focused on investigating the applicability range and potential limitations of the presented technique. In particular, it will be applied to test cases featuring larger number of design variables, as well as a larger number of operating frequencies (three and four). It is expected that for increased problem complexity certain numerical issues, e.g., associated with identification of the scaling directions and their actual effects on the operating parameters might limit the method’s efficacy. At the same time, it is planned to carry out extended benchmarking involving both local and global optimization methods.

## Data Availability

The datasets generated during and/or analysed during the current study are available from the corresponding author on reasonable request.
